# Pediatric-type high-grade neuroepithelial tumors with *CIC* gene fusion share a common DNA methylation signature

**DOI:** 10.1038/s41698-023-00372-1

**Published:** 2023-03-24

**Authors:** Philipp Sievers, Martin Sill, Daniel Schrimpf, Zied Abdullaev, Andrew M. Donson, Jessica A. Lake, Dennis Friedel, David Scheie, Olli Tynninen, Tuomas Rauramaa, Kaisa L. Vepsäläinen, David Samuel, Rebecca Chapman, Richard G. Grundy, Kristian W. Pajtler, Arnault Tauziède-Espariat, Alice Métais, Pascale Varlet, Matija Snuderl, Thomas S. Jacques, Kenneth Aldape, David E. Reuss, Andrey Korshunov, Wolfgang Wick, Stefan M. Pfister, Andreas von Deimling, Felix Sahm, David T. W. Jones

**Affiliations:** 1 Department of Neuropathology, Institute of Pathology, University Hospital Heidelberg, Heidelberg, Germany; 2 Clinical Cooperation Unit Neuropathology, German Consortium for Translational Cancer Research (DKTK), German Cancer Research Center (DKFZ), Heidelberg, Germany; 3 Hopp Children’s Cancer Center Heidelberg (KiTZ), Heidelberg, Germany; 4 Division of Pediatric Neurooncology, German Cancer Consortium (DKTK), German Cancer Research Center (DKFZ), Heidelberg, Germany; 5 Laboratory of Pathology, Center for Cancer Research, National Cancer Institute, National Institutes of Health, Bethesda, MD USA; 6 Morgan Adams Foundation Pediatric Brain Tumor Research Program, Children’s Hospital Colorado, Aurora, CO USA; 7 Department of Pediatrics, University of Colorado Anschutz Medical Campus, Aurora, CO USA; 8 Center for Cancer and Blood Disorders, Children’s Hospital Colorado, Aurora, CO USA; 9 Department of Pathology, Rigshospitalet, Copenhagen University Hospital, Copenhagen, Denmark; 10 Department of Pathology, HUSLAB, University of Helsinki and Helsinki University Hospital, Helsinki, Finland; 11 Department of Pathology, Kuopio University Hospital, University of Kuopio, Kuopio, Finland; 12 Unit of Pathology, Institute of Clinical Medicine, University of Eastern Finland, Kuopio, Finland; 13 Department of Pediatrics, Kuopio University Hospital, University of Kuopio, Kuopio, Finland; 14 Department of Hematology/Oncology, Valley Children’s Hospital, Madera, CA USA; 15 Children’s Brain Tumour Research Centre, University of Nottingham, Nottingham, UK; 16 Department of Pediatric Oncology, Hematology, Immunology and Pulmonology, University Hospital Heidelberg, Heidelberg, Germany; 17 Department of Neuropathology, GHU Paris—Psychiatry and Neuroscience, Sainte-Anne Hospital, Paris, France; 18 Institut de Psychiatrie et Neurosciences de Paris (IPNP), UMR S1266, INSERM, IMA-BRAIN, Paris, France; 19 Department of Pathology, NYU Langone Medical Center, New York, NY USA; 20 Developmental Biology and Cancer Research and Teaching Department, UCL Great Ormond Street Institute of Child Health, London, UK; 21 Department of Histopathology, Great Ormond Street Hospital for Children NHS Foundation Trust, London, UK; 22 Clinical Cooperation Unit Neurooncology, German Consortium for Translational Cancer Research (DKTK), German Cancer Research Center (DKFZ), Heidelberg, Germany; 23 Department of Neurology and Neurooncology Program, National Center for Tumor Diseases, Heidelberg University Hospital, Heidelberg, Germany; 24 Division of Pediatric Glioma Research, German Cancer Research Center (DKFZ), Heidelberg, Germany

**Keywords:** CNS cancer, Molecular medicine

## Abstract

Pediatric neoplasms in the central nervous system (CNS) show extensive clinical and molecular heterogeneity and are fundamentally different from those occurring in adults. Molecular genetic testing contributes to accurate diagnosis and enables an optimal clinical management of affected children. Here, we investigated a rare, molecularly distinct type of pediatric high-grade neuroepithelial tumor (*n* = 18), that was identified through unsupervised visualization of genome-wide DNA methylation array data, together with copy number profiling, targeted next-generation DNA sequencing, and RNA transcriptome sequencing. DNA and/or RNA sequencing revealed recurrent fusions involving the *capicua transcriptional repressor* (*CIC*) gene in 10/10 tumor samples analyzed, with the most common fusion being *CIC::LEUTX* (*n* = 9). In addition, a *CIC::NUTM1* fusion was detected in one of the tumors. Apart from the detected fusion events, no additional oncogenic alteration was identified in these tumors. The histopathological review demonstrated a morphologically heterogeneous group of high-grade neuroepithelial tumors with positive immunostaining for markers of glial differentiation in combination with weak and focal expression of synaptophysin, CD56 and CD99. All tumors were located in the supratentorial compartment, occurred during childhood (median age 8.5 years) and typically showed early relapses. In summary, we expand the spectrum of pediatric-type tumors of the CNS by reporting a previously uncharacterized group of rare high-grade neuroepithelial tumors that share a common DNA methylation signature and recurrent gene fusions involving the transcriptional repressor *CIC*. Downstream functional consequences of the fusion protein *CIC::LEUTX* and potential therapeutic implications need to be further investigated.

## Introduction

Pediatric neoplasms in the central nervous system (CNS) are extremely heterogeneous and diagnostically challenging tumors. According to data from the Central Brain Tumor Registry of the United States (CBTRUS), CNS tumors have become the leading cause of cancer-related death in childhood^1^. Accurate diagnosis is crucial for an optimal management of children with these diseases. During the last several years, remarkable advances in our understanding of the molecular underpinnings of these tumors have occurred as a result of comprehensive (epi-)genetic profiling and led to substantial progress in the classification and therapy of pediatric CNS tumors^2^. In addition, several novel and extremely rare tumor types have been identified using state-of-the-art molecular methods such as DNA methylation arrays and genetic profiling^3^. More recently, a wide range of different oncogenic gene fusions outside the mitogen-activated protein kinase (MAPK) pathway have shown to play an important role in driving tumorigenesis of pediatric CNS tumors^4–6^, some of them with the potential to provide novel therapeutic options.

DNA methylation profiling of CNS tumors has been demonstrated to be a powerful tool for molecular tumor classification with the additional evaluation of copy number profiles being extremely useful for the identification of oncogenic gene fusions^4,7–9^. Such an approach is particularly valuable for the discovery and characterization of rare and novel tumor types that show a wide variety of clinicopathological appearances^5^.

Here, we describe a novel molecular CNS tumor type, primarily occurring in children, identified through unsupervised visualization of a large cohort of genome-wide DNA methylation profiling data, together with targeted next-generation DNA sequencing, and RNA transcriptome sequencing.

## Results

### DNA methylation profiling reveals an epigenetically distinct group of pediatric-type neuroepithelial tumors

**Fig. 1:**
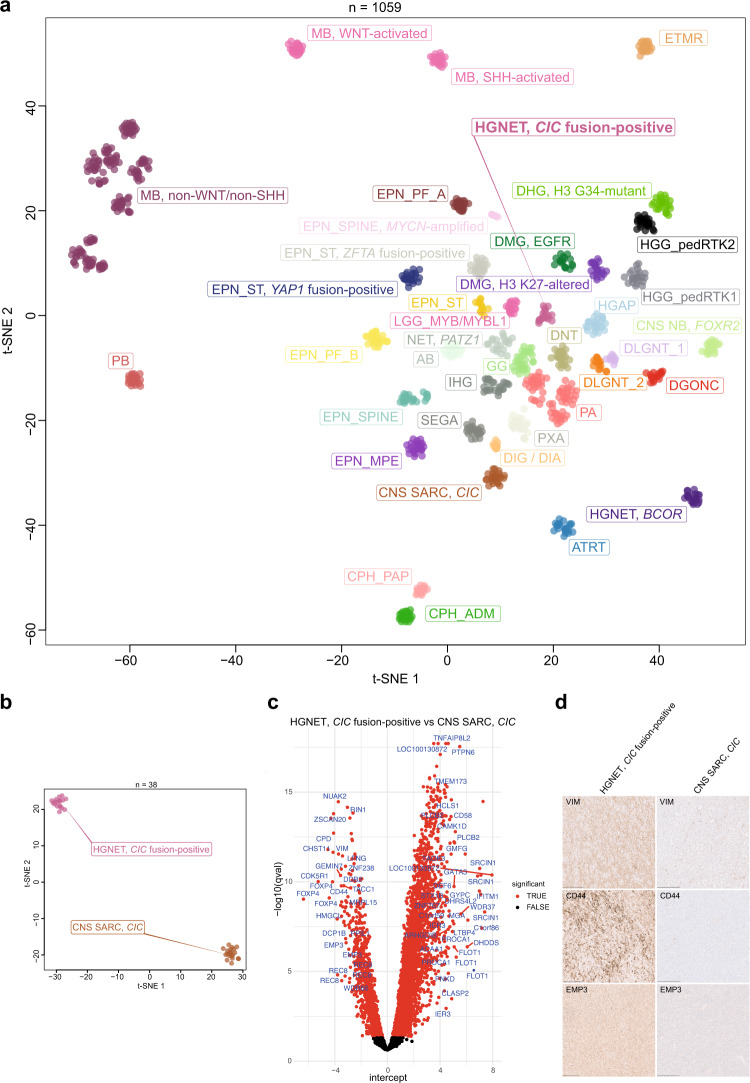
Molecular classification of high-grade neuroepithelial tumors *CIC* fusion-positive by DNA methylation profiling. **a** Unsupervised, nonlinear t-distributed stochastic neighbor embedding (t-SNE) projection of DNA methylation array profiles from 1059 tumors. DNA methylation profiling reveals a molecular distinct group of high-grade neuroepithelial tumors (HGNET, *CIC* fusion-positive; *n* = 18). **b** t-SNE analysis of DNA methylation array profiles of HGNET, *CIC* fusion-positive, and *CIC*-rearranged sarcoma (CNS SARC, *CIC*). For DNA methylation class abbreviations, see Supplementary Table 2. **c** Volcano plot comparing differentially methylated probes between HGNET, *CIC* fusion-positive and CNS SARC, *CIC*. **d** Immunohistochemical expression of vimentin (VIM), CD44, and EMP3 in HGNET, *CIC* fusion-positive and CNS SARC, *CIC*. Scale bars 300 μm.

**Fig. 2:**
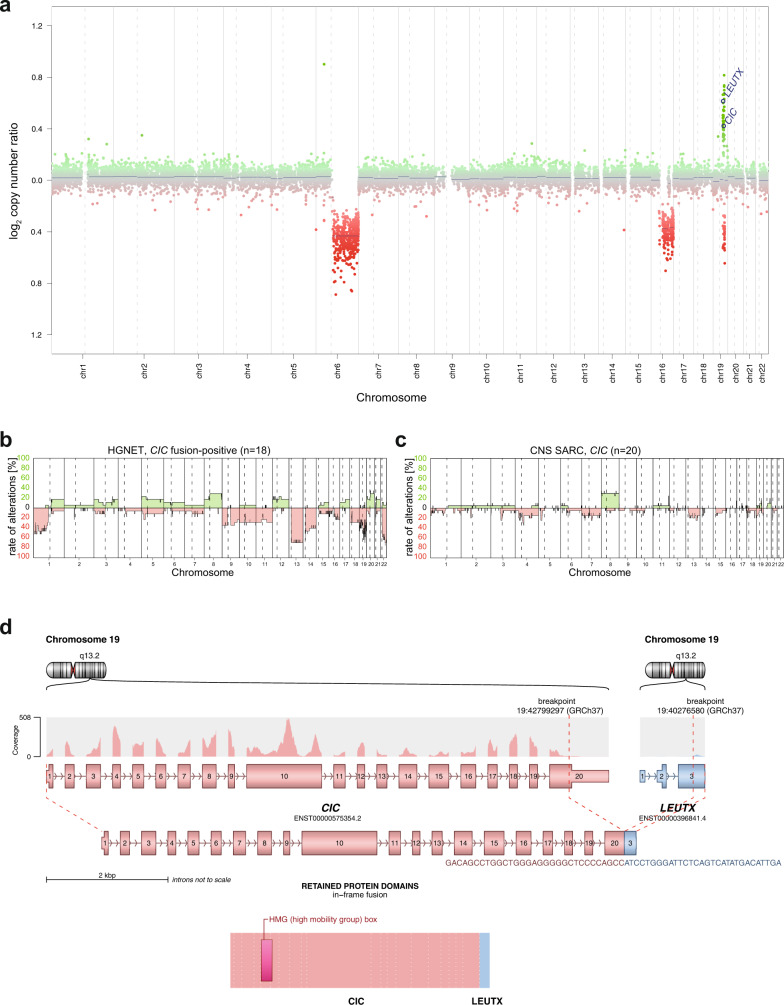
Molecular characteristics of high-grade neuroepithelial tumors *CIC* fusion-positive (HGNET, *CIC* fusion-positive). **a** Copy number profile derived from DNA methylation array data of a HGNET, *CIC* fusion-positive showing structural alterations affecting chromosome 19q around the *CIC* and *LEUTX* locus. **b**, **c** Summary plot of copy number alterations in HGNET, *CIC* fusion-positive and *CIC*-rearranged sarcoma (CNS SARC, *CIC*). **d** Visualization of the *CIC::LEUTX* gene fusion detected by RNA sequencing, in which exons 1–20 of *CIC*, as the 5′ partner, are fused to exon 3 of *LEUTX*.

Through unsupervised visualization of genome-wide DNA methylation data from a large cohort of approximately 90,000 pediatric and adult CNS tumor samples, we identified an epigenetically distinct group of tumors (*n* = 16), that did not match any known DNA methylation class. This group was comprised of tumors with a wide spectrum of original histological diagnoses, including predominantly high-grade glioma (such as glioblastoma, anaplastic astrocytoma, or ganglioglioma), with many tumors considered as not classifiable or with a descriptive diagnosis (Supplementary Table 1). In addition, two further CNS tumor samples harboring a *CIC::LEUTX* fusion (see below) that have already been published were included into subsequent molecular profiling^10^. A more selected visualization (t-SNE) of DNA methylation patterns of tumors in this novel cluster compared with well-characterized reference samples (tumor samples included in the current version of the Heidelberg DNA methylation brain tumor classifier with a calibrated score >0.9; Supplementary Table 2) confirmed a clearly distinct grouping (Fig. 1a). Importantly, no similarity was seen with the recently described tumor type “*CIC*-rearranged sarcoma” (previously CNS Ewing sarcoma family tumor with *CIC* alteration; Fig. 1b)^11^. Further analysis of differentially methylated regions between tumors within the novel group and *CIC*-rearranged sarcoma showed aberrant methylation patterns, including promoter region hypomethylation amongst others of *CD44*, *EMP3*, and *VIM* in these tumors (Fig. 1c). This was supported by an inverse expression profile of the respective markers by immunohistochemistry (*n* = 4; Fig. 1d). Analysis of copy number profiles derived from the raw intensities of the DNA methylation array probes revealed recurrent structural aberrations on chromosome 19q around the genetic loci of the *capicua* transcriptional repressor (*CIC*) and *leucine twenty homeobox* (*LEUTX*) in all samples (Fig. 2a and Supplementary Fig. 1). Further recurrent copy number alterations included: loss of chromosome 1p, 13q, 14q, and 22q (Fig. 2b). Gain of chromosome 8, typically present in *CIC*-rearranged sarcoma (Fig. 2c), was seen in a high proportion of cases as well (Fig. 2b). A summary of detected structural aberrations is given in Supplementary Table 3.

### *CIC* gene rearrangements are a characteristic feature of tumors within the novel group

By targeted next-generation DNA sequencing and/or RNA sequencing, 9 out of 10 tumors analyzed (including the two previously published samples) demonstrated gene fusions between *CIC* and *leucine twenty homeobox* (*LEUTX*) as the 3′ partner, both located on chromosome 19q13.2 (Fig. 2d). In all of the tumors, exons 1–20 of *CIC* (NM_015125.5) were fused in frame to exon 3 of *LEUTX* (NM_001143832.2), retaining the DNA-binding high-mobility group (HMG) box of CIC and the suggested 9aaTAD domain of *LEUTX*^12^. These findings are in line with the breakpoints detected in a previously reported pediatric embryonal tumor of the CNS^13^ (Supplementary Table 4). In addition, a fusion between exons 1–20 of *CIC* and *NUT midline carcinoma family member 1* (*NUTM1*, located on chromosome 15q14) exons 2–7 (NM_001284293.1) was observed, very similar to the *CIC::LEUTX* rearrangement (Supplementary Fig. 2 and Supplementary Table 4). Apart from the detected fusion events, no additional oncogenic alteration was identified in these tumors based on sequencing.

### Clinical characteristics and morphological features indicate pediatric‐type high-grade neuroepithelial tumors

**Fig. 3:**
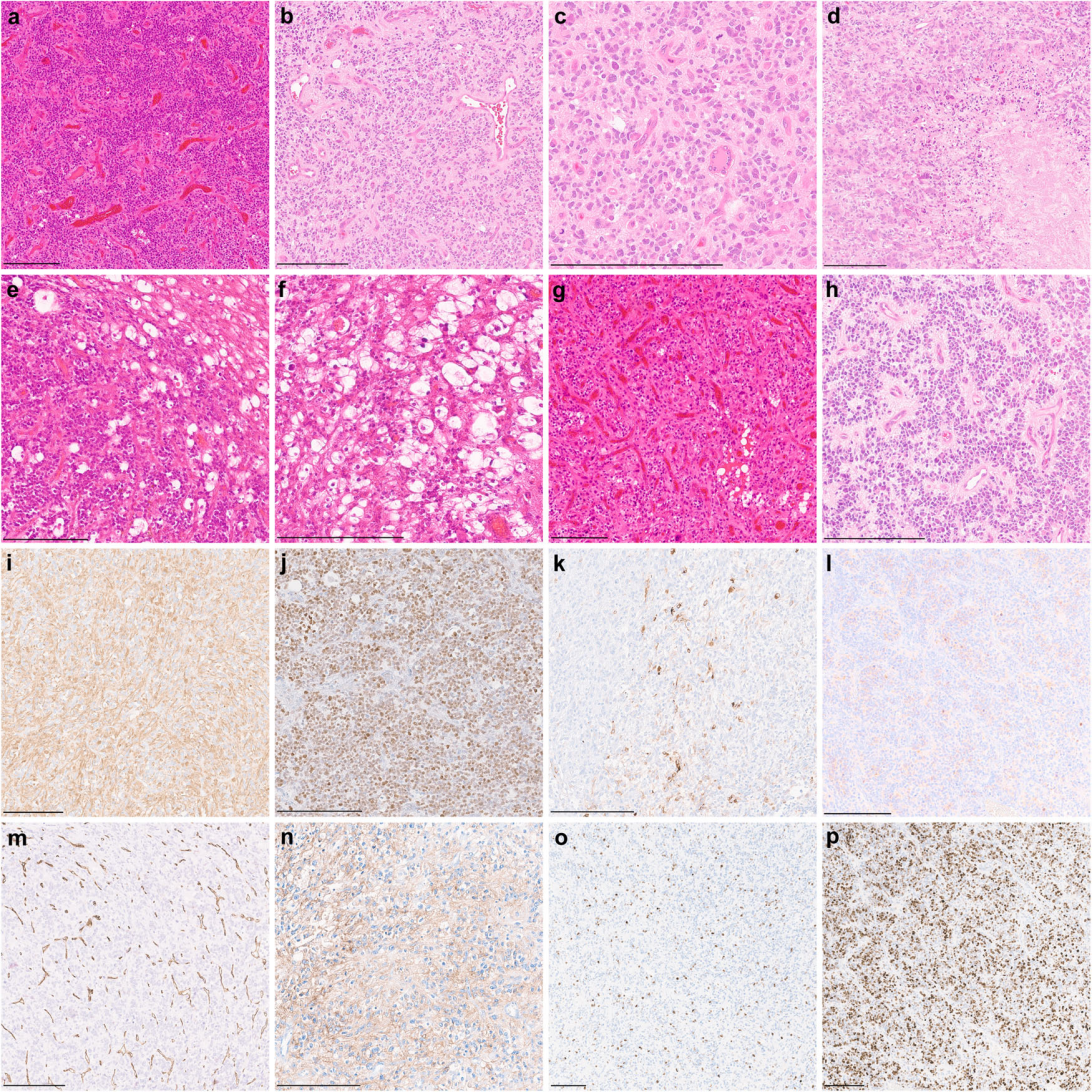
Morphological and immunohistochemical features of high-grade neuroepithelial tumors *CIC* fusion-positive. **a** Histologically, tumors show a high increase in cellular density of slightly pleomorphic neoplastic cells. **b**, **c** A more pronounced cellular pleomorphism with multinucleated cells is present in a subset of cases. **d** Tumor necrosis. **e**, **f** An oligodendroglial morphology with perinuclear halos is focally present in a minor proportion of tumors. **g**, **h** Tumors are highly vascularized with a subset of cases demonstrating perivascular anucleate zones (pseudorosettes). **i**, **j** Positive immunostaining for markers of glial differentiation (GFAP and OLIG2). **k**, **l** Tumor cells show focal immunoreactivity for MAP2 and synaptophysin. **m** CD34 expression is restricted to the vessels. **n** CD99 expression is focally present in all evaluated samples. **o,** **p** Ki-67 labeling indices range from about 10 to 70% of the neoplastic cells. Scale bars 200 μm.

Analysis of available clinical data demonstrated that all tumors were located in the supratentorial compartment, mainly in the parietal and occipital lobe. The median age at presentation was 8.5 years (range 1–19) and the sex distribution was not significantly biased when considering the small number of patients. Clinical outcome data were available for only six patients. Median PFS was 13.5 months (range 6–16 months) with all of the patients experiencing a relapse during the follow-up period. Only one of the patients died of the disease during the follow-up period at 15 months after diagnosis. Together, these initial data suggest an intermediate malignancy grade (Supplementary Fig. 3). Initial histopathologic diagnoses comprised various tumor types of mainly high-grade glioma. More detailed descriptions of the cases are given in Supplementary Table 1. A histopathological review was performed on a subset of the tumors with available material (*n* = 9) that revealed a morphologically heterogeneous group. Histologically, all reviewed tumors shared a high cellular density with most neoplasms showing slightly pleomorphic neoplastic cells often with remarkably condensed chromatin (Fig. 3a). A more pronounced cellular pleomorphism with multinucleated cells were seen in single cases (Fig. 3b–d). An oligodendrocyte-like phenotype with perinuclear clearing was focally found in four of the cases (Fig. 3e, f). Microcystic changes were present in half of the tumors. Tumors were highly vascularized with hypertrophic or proliferated vessels in most of the cases (Fig. 3g). In three of the tumors, perivascular anucleate zones (pseudorosettes) were observed (Fig. 3h). Necrosis was present in five tumor samples (Fig. 3d). Mitotic activity was generally high, with the exception of one case. Immunostaining for markers of glial differentiation (GFAP and OLIG2) was positive in all tumors (Fig. 3i, j). However, GFAP expression was only weakly positive or restricted to a minor proportion of neoplastic cells in some of the cases. In 4/4 tumors, a focal immunoreactivity for MAP2 was detected (Fig. 3k). All tumors showed a weak and focal positivity for synaptophysin (*n* = 9; Fig. 3l). CD56 was expressed in all samples analyzed (*n* = 4). All evaluated tumors had absent immunostaining for NeuN (*n* = 5). CD34 (*n* = 6) expression was restricted to the vessels (Fig. 3m). A focal positivity for CD99 was observed in all evaluated samples (*n* = 4; Fig. 3n). Ki-67 labeling indices ranged from 10 to 70% (Fig. 3o, p).

## Discussion

Here, we describe a previously uncharacterized group of rare, pediatric CNS tumors that was discovered through unsupervised visualization of genome-wide DNA methylation profiles. This novel group of tumors, epigenetically distinct from all known CNS neoplasms, shows recurrent gene fusions involving the transcriptional repressor *CIC* (most commonly with *LEUTX*) as an additional unifying feature.

*CIC*, a human homolog of *capicua* in Drosophila, acts as a transcriptional repressor with a DNA-binding high-mobility group (HMG) box domain that normally inhibits *ETV1/4/5* expression and counteracts activation of genes downstream of receptor tyrosine kinase (RTK) signaling^14^. Aberrations in *CIC* have been identified in various types of cancer, with loss-of-function mutations frequently observed in oligodendroglioma^15,16^, leading to activation of downstream RTK signaling. Intriguingly, rearrangements involving *CIC* and the double homeobox 4 (*DUX4*) commonly found in high-grade round cell undifferentiated sarcoma^17,18^, have been shown to enhance the transcriptional activity of *CIC* downstream targets, including the member of the ETS family of transcription factors, such as *ETV1/4/5*^19,20^. Consistent with that, an upregulation of members of the ETS transcription factor family have been reported in *CIC*-rearranged sarcoma (“Ewing sarcoma family of tumors with *CIC* alterations”) harboring oncogenic fusions between *CIC* and *NUTM1*^11^.

In contrast, *LEUTX* is a member of the paired (PRD)-like homeobox gene family of transcription factors and is expressed almost exclusively in early embryos where it is thought to play a role during preimplantation development^21,22^. Although rearrangements between *CIC* and *LEUTX* have been reported recently^10,23^, the exact role in tumorigenesis of *LEUTX* remains poorly defined. However, since *LEUTX* and *DUX4* belong to the same class of paired (PRD)-like homeobox genes an oncogenic mechanism very similar to that of the *CIC::DUX4* chimeric transcript seems very likely. Downstream functional consequences of this novel fusion protein and potential therapeutic implications need to be further investigated.

Although both previously described *CIC::LEUTX*-fused CNS tumor samples^10^, that were now included into the present series, also clustered with this novel epigenetic group, it seems still too early at this point to say whether this alteration is specific enough to use it as an “essential diagnostic criterion” according to the recently published fifth edition of the World Health Organization (WHO) classification of CNS tumors^24^. The same considerations also apply to the histomorphological and immunohistochemical findings that demonstrated a morphologically heterogeneous group of high-grade neuroepithelial tumors with positive immunostaining for markers of glial differentiation in combination with weak and focal expression of synaptophysin and CD99. High expression of CD44, EMP3 and VIM may be useful to distinguish the tumors from *CIC*-rearranged sarcoma. However, this will need further validation. Thus, we suggest the specific epigenetic signature by DNA methylation profiling as so far the only method to accurately identify these tumors. Although there are strong arguments for consideration of these tumors as a specific type of glioma (typical glioma- and/or ependymoma-like features in combination with consistent expression of GFAP and OLIG2), the lack of a clear indication of a particular lineage and the small number of cases with sufficient material for a comprehensive histopathological evaluation suggest the provisionally use of the term “neuroepithelial” to describe these neoplasms. *CIC* fusion-positive neuroepithelial tumors may be considered for inclusion into upcoming classifications of CNS tumors to help diagnose these tumors more precisely. This would be either possible as a provisional tumor type in the category “gliomas, glioneuronal tumors, and neuronal tumors” or within a new “molecular” category of tumors with currently unclear lineage. In addition, more clinical workup in terms of patient outcome data is urgently needed to characterize these neoplasms in more detail. Our initial follow-up data suggest an intermediate malignancy grade with all tumors showing early relapses. However, it seems like maybe whatever second-line therapies were applied are more effective, with only one death and one longer survivor despite initial quick relapse.

In summary, we expand the spectrum of pediatric-type tumors of the CNS by reporting a previously uncharacterized group of rare high-grade neuroepithelial tumors that share a common DNA methylation signature and recurrent gene fusion involving *CIC*. These findings also imply a growing biological understanding of the genomics underlying rare pediatric CNS tumors. Given their broad morphological spectrum and recurrent gene fusions involving *CIC*, we suggest the term ‘high-grade neuroepithelial tumor *CIC* fusion-positive’ to describe this novel tumor type.

## Methods

### Sample collection

Patient tumor samples and retrospective clinical information were provided by multiple national and international collaborating centers and collected at the Department of Neuropathology of the University Hospital Heidelberg (Heidelberg, Germany) and German Cancer Research Center (DKFZ, Heidelberg, Germany). Sample selection was based on unsupervised visualization (t-distributed stochastic neighbor embedding (t-SNE) and uniform manifold approximation and projection (UMAP)) of genome-wide DNA methylation array data that revealed a molecularly distinct group of tumors forming a cluster separate from all established tumor types. In addition, two further CNS tumor samples harboring a *CIC::LEUTX* fusion that have already been published were included into subsequent molecular profiling^10^. Furthermore, DNA methylation array data of numerous well-characterized reference samples included in the most recent classifier version (v12.5) representing CNS tumors were used for comparative analyses. Detailed descriptions of the reference DNA methylation classes are outlined under (https://www.molecularneuropathology.org). This study is covered by the ethical approval of the University of Heidelberg medical faculty (ethical vote S-318/2022) which confirmed that written consent specific for this study can be waived since it is based on archival material that was remaining after regular diagnostic workup. Clinical details of the patients are listed in Supplementary Table 1.

### Histology and immunohistochemistry

For a subset of samples (*n* = 9), a histopathological review was retrospectively performed to investigate the morphological and immunohistochemical features of tumors within the novel group. Due to the aspect of a multicenter cohort, the availability of tissue was restricted for some of the cases. Hematoxylin and eosin (H&E) and immunohistochemical staining was either performed at the Department of Neuropathology of the University Hospital Heidelberg or received from the respective collaborator institutes. Immunohistochemical staining was performed on a Ventana BenchMark ULTRA Immunostainer using the ultraView Universal DAB Detection Kit (Ventana Medical Systems, Tucson, AZ, USA). Antibodies were directed against: glial fibrillary acid protein (GFAP, Z0334, rabbit polyclonal, 1:1000 dilution, Dako Agilent, Santa Clara, CA, USA), oligodendrocyte lineage transcription factor 2 (OLIG2, clone EPR2673, rabbit monoclonal, 1:50 dilution, Abcam, Cambridge, UK), MAP2 (clone HM-2, mouse monoclonal, 1:15000 dilution, Sigma-Aldrich, St. Louis, MO, USA), Synaptophysin (clone MRQ-40, rabbit monoclonal, 1:160 dilution, Cell Marque Corp., Rocklin, CA, USA), NeuN (clone A60, mouse monoclonal, 1:100 dilution, Millipore, Burlington, MA, USA), CD56 (clone MRQ-42, rabbit monoclonal, 1:800 dilution, Cell Marque Corp.), CD34 (clone QBEnd/10, mouse monoclonal, Ventana Medical Systems), CD99 (CONFIRM anti-CD99, O13, mouse, monoclonal, Roche, Basel, Switzerland), Ki-67 (clone MIB-1, mouse monoclonal, 1:100 dilution, Dako Agilent), CD44 (156-3C11, mouse monoclonal, 1:50 dilution, Cell Signaling, Danvers, MA, USA), vimentin (clone V9, mouse monoclonal, 1:900 dilution, Dako Agilent) and EMP3 (clone158/8, mouse monoclonal, 1:5 dilution, DKFZ^25^).

### DNA and RNA extraction

Tumor DNA and RNA of samples processed in Heidelberg were extracted from areas with highest tumor cell content using the automated Maxwell system (Promega, Madison, WI, USA). Genomic DNA was extracted from fresh frozen or formalin-fixed and paraffin-embedded (FFPE) tissue samples with the Maxwell 16 Tissue DNA Purification Kit or the Maxwell 16 FFPE Plus LEV DNA Purification Kit (Promega), according to the manufacturer’s instructions. RNA was extracted from FFPE tissue samples by following the Maxwell 16 LEV RNA FFPE Kit protocol (Promega). Nucleic acid concentrations were determined using the Invitrogen Qubit dsDNA BR Assay Kit (Thermo Fisher Scientific, Waltham, MA, USA) on a FLUOstar Omega Microplate Reader (BMG Labtech, Ortenberg, Germany). For single cases, DNA was extracted using either the AllPrep DNA/RNA FFPE Kit (Qiagen, Hilden, Germany), QIAamp DNA Micro Kit (Qiagen) or FormaPure Kit (Beckman Coulter, Brea, CA, USA), according to the manufacturer’s instructions.

### DNA methylation array processing and copy number profiling

Genome-wide DNA methylation profiling of all samples was performed using the Infinium MethylationEPIC (EPIC) BeadChip (Illumina, San Diego, CA, USA) or Infinium HumanMethylation450 (450k) BeadChip array (Illumina) according to the manufacturer’s instructions and as previously described^9^. Raw data were generated at the Department of Neuropathology of the University Hospital Heidelberg, the Genomics and Proteomics Core Facility of the DKFZ or at respective international collaborator institutes, using both fresh–frozen and FFPE tissue samples. All computational analyses were performed in R version 4.6.1 (R Development Core Team, 2020; https://www.R-project.org). Copy number variation analysis from 450k and EPIC methylation array data was performed using the conumee Bioconductor package version 1.12.0. Summary copy number profiles to display rates of copy number gains and losses per DNA methylation class were generated using an in-house R script (https://github.com/dstichel/CNsummaryplots). Focal copy number alterations were called based on manual review of the log2 ratio plots for each sample. Raw signal intensities were obtained from IDAT-files using the minfi Bioconductor package version 1.21.4^26^. Illumina EPIC and 450k samples were merged to a combined data set by selecting the intersection of probes present on both arrays (combineArrays function, minfi). Each sample was individually normalized by performing a background correction (shifting of the 5% percentile of negative control probe intensities to 0) and a dye-bias correction (scaling of the mean of normalization control probe intensities to 10,000) for both color channels. Subsequently, a correction for the array type (450k/EPIC) was performed by fitting univariable, linear models to the log2-transformed intensity values (removeBatchEffect function, limma package version 3.30.11). The methylated and unmethylated signals were corrected individually. Beta-values were calculated from the retransformed intensities using an offset of 100 (as recommended by Illumina). All samples were checked for duplicates by pairwise correlation of the genotyping probes on the 450k/EPIC array. To perform unsupervised nonlinear dimension reduction, the remaining probes after standard filtering^9^ were used to calculate the 1-variance weighted Pearson correlation between samples. The resulting distance matrix was used as input for t-SNE analysis (Rtsne package version 0.13). The following non-default parameters were applied: is_distance = T, theta = 0, pca = F, max_iter = 10,000, perplexity = 30. Estimation of differential methylated positions (DMP) was done in R by using the function “dmpFinder” from the minfi package (v1.43). The Illumina EPIC platform was used to annotate CpGs by their position in the genome and associated genes. The tests were carried out on the M-values of all promoter-associated genes as well as the top 100k CpGs according to mean average deviance. CpGs with an FDR *q*-value smaller than 0.05 were considered as significant differential methylated. Volcano plots of the DMPs were generated by the R-package ggplot2 (v3.3.6). CpGs are distributed according to their −log10 *Q* values and fold change (intersect). Further significant different methylated CpGs are depicted in red while the associated gene top100 DMP is shown.

### Targeted next-generation DNA sequencing

For a subset of samples with DNA available (*n* = 9), DNA sequencing using a customized enrichment/hybrid-capture-based next-generation sequencing (NGS) gene panel were performed on a NextSeq 500 or NovaSeq 6000 instrument (Illumina) at the Department of Neuropathology of the University Hospital Heidelberg (Heidelberg, Germany)^27^. The NGS panel comprised the entire coding (all exons + /– 25 bp) and selected intronic and promoter regions of 170 genes of particular relevance in CNS tumors, and was designed to detect single nucleotide variants (SNV), small insertions/deletions (InDel), exonic rearrangements, and recurrent fusion events. Paired-end sequencing was applied to increase the detection sensitivity of duplicates and possible gene fusions. Sequence reads were mapped to the reference human genome build GRCh37 (hg19) using the Burrows–Wheeler aligner (BWA).

### RNA sequencing and analysis

RNA sequencing for the purpose of gene fusion detection of samples for which RNA of sufficient quality and quantity was available (*n* = 8) was performed as previously described^28^. In brief, RNA sequencing libraries were prepared using the TruSeq RNA Library Prep for Enrichment kit (Illumina) and paired-end reads were sequenced on a NextSeq 500 or NovaSeq 6000 instrument (Illumina). After adapter trimming, reads were aligned to the human genome (GRCh37) with the STAR aligner^29^ and counted using RSEM^30^. Fastq files from transcriptome sequencing were used for de novo annotation of fusion transcripts using the Arriba (v1.2.0) algorithm^31^ with standard parameters, which removes recurrent alignment artifacts, transcript variants also observed in normal tissue, reads with low sequence complexity, and events with short anchors or breakpoints in close proximity or a low number of supporting reads relative to the overall number of predicted events in a gene.

### Survival analysis

Survival analysis was performed using GraphPad Prism 9 (GraphPad Software, La Jolla, CA, USA). Data on survival could be retrospectively retrieved for six patients. Overall survival (OS) and progression-free survival (PFS) probabilities were displayed using the Kaplan–Meier method.

### Reporting summary

Further information on research design is available in the Nature Research Reporting Summary linked to this article.

## Supplementary information

Supplementary Information

Supplementary Tables

Reporting Summary

## Data Availability

DNA methylation data generated during this study has been deposited in NCBIs Gene Expression Omnibus (GEO, http://www.ncbi.nlm.nih.gov/geo) under accession number GSE223546. DNA methylation data used as a reference has been deposited under accession number GSE90496. Consent for public data sharing of sequencing data was not obtained from the patients, so datasets are available upon IRB-approved collaboration from the corresponding author (PS) and will only be shared for research-related, non-commercial purposes. The remaining data are available within the article and supplementary material.

## Code availability

R packages and scripts used to analyze the data, along with input data, are explained in the methods and supplementary material. The code underlying random forest-based algorithm for DNA methylation-based CNS tumor classification was previously described^9^.
