# Supplementary material for: Pediatric-type high-grade neuroepithelial tumors with CIC gene fusion share a common DNA methylation signature
Source: NPJ Precis Oncol. 2023 Mar 24;7:30. doi: 10.1038/s41698-023-00372-1 (PMC10039012; doi:10.1038/s41698-023-00372-1)
Supplement: Supplementary file 1 — Supplementary Information [file 41698_2023_372_MOESM1_ESM.pdf]

Supplemental Information

Pediatric-type high-grade neuroepithelial tumors with *CIC* gene fusion share a common DNA methylation signature

Philipp Sievers, Martin Sill, Daniel Schrimpf, Zied Abdullaev, Andrew M. Donson, Jessica A. Lake, Dennis Friedel, David Scheie, Olli Tynninen, Tuomas Rauramaa, Kaisa L. Vepsäläinen, David Samuel, Rebecca Chapman, Richard G. Grundy, Kristian W. Pajtler, Arnault Tauziède-Espariat, Alice Métais, Pascale Varlet, Matija Snuderl, Thomas S. Jacques, Kenneth Aldape, David E. Reuss, Andrey Korshunov, Wolfgang Wick, Stefan M. Pfister, Andreas von Deimling, Felix Sahm, David T. W. Jones

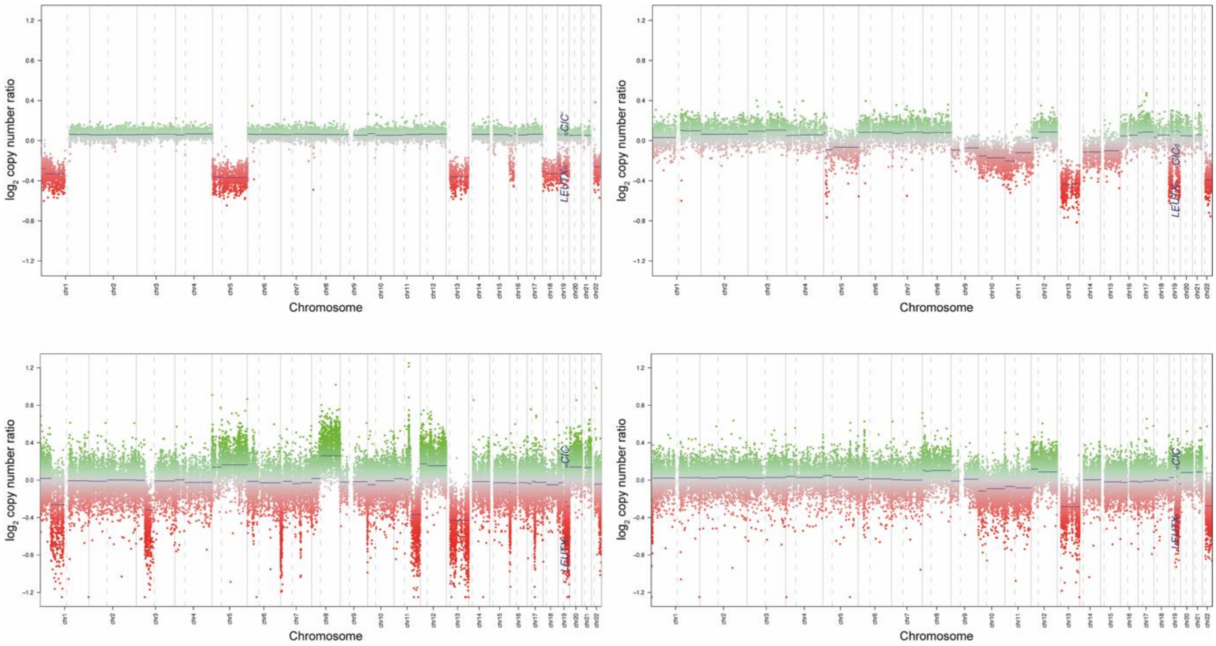

Supplementary Figure 1 Copy number profiles derived from DNA methylation array data of HGNETs *CIC* fusion-positive. Copy number variation (CNV) plots of four different HGNETs *CIC* fusion-positive showing recurrent structural alterations affecting chromosome 19q around the *CIC* and *LEUTX* locus.

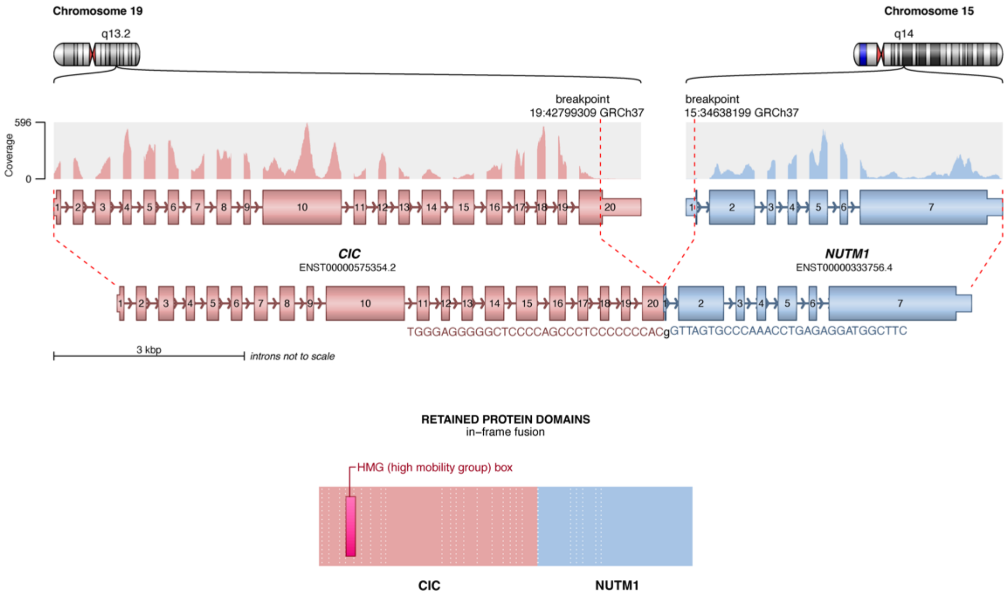

Supplementary Figure 2 Illustration of the *CIC::NUTM1* gene fusion. Visualization of the *CIC::NUTM1* fusion detected by RNA sequencing using the ‘Arriba’ algorithm.

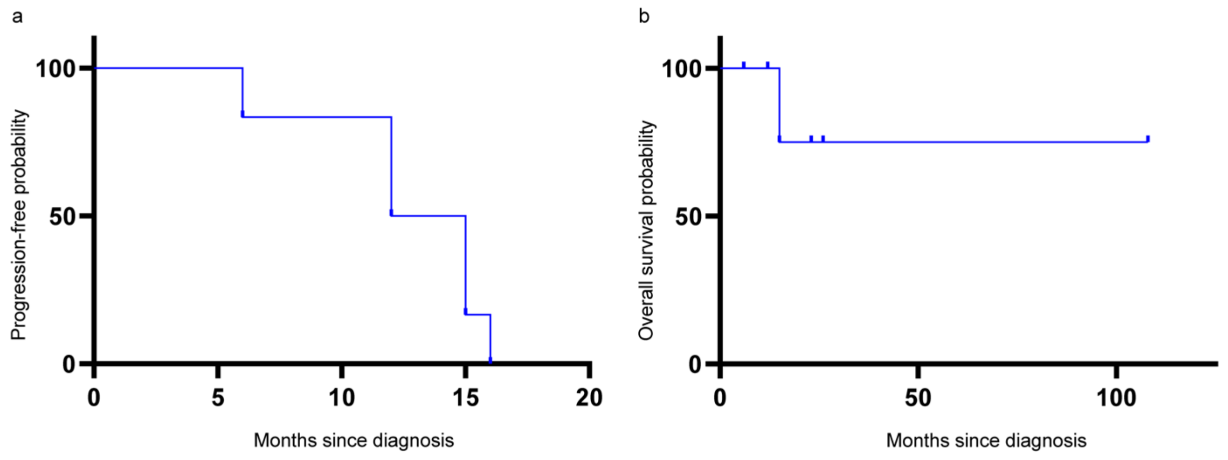

Supplementary Figure 3 Clinical outcomes of patients with HGNET *CIC* fusion-positive. a, Kaplan-Meier curve for progression-free survival of six patients from the investigated cohort for whom outcome data was available. b, Kaplan-Meier curve for overall survival of patients (n = 6) from the investigated cohort.

tSNE analysis

tSNE analysis

The tSNE analysis was done with the following packages and settings.

The used R version was: R version 4.0.3 (2020-10-10) – "Bunny-Wunnies Freak Out"

During the analysis the following packages are used:

```
library(ggplot2)
library(ggthemes)
library(plyr)
library(Rtsne)
```

Preprocessing

Loading the methylation data

```
beta_values.GPL13534 <- read.table('GPL13534_matrix_processed.txt', sep='\t', header=TRUE)
beta_values.GPL13534 <- beta_values.GPL13534[,!grepl('Detection',
                                                      colnames(beta_values.GPL13534))]

beta_values.GPL21145 <- read.table('GPL21145_matrix_processed.txt',sep='\t', header=TRUE)
beta_values.GPL21145 <- beta_values.GPL21145[,!grepl('Detection',
                                                      colnames(beta_values.GPL21145))]

betas.tmp <- cbind(beta_values.GPL13534, beta_values.GPL21145[,-1])

betas <- data.matrix(betas.tmp[,-1])
rownames(betas) <- betas.tmp$ID_REF
colnames(betas) <- gsub('X','', colnames(betas))
```

Loading the annotation data

```
annotation_data <- read.table('annotation.txt', sep='\t', header=TRUE)

groups <- levels(as.factor(annotation_data$group))
colors <- unlist(lapply(groups,
                        function(group) {
                          annotation_data$group_color[annotation_data$group == group][1]
                        }))

annotations <- data.frame(
  txt_idat=annotation_data$idat,
  sampleName=annotation_data$sample,
  GROUP=annotation_data$group_color
)
```

```
legends <- list(
  group=list(names=rev(groups), colors=rev(colors))
)
```

Selection of beta values

```
betaCriteria.table <- apply(betas,1,sd)
selected.cpgs <- sort(betaCriteria.table, decreasing=TRUE)[1:8000]
beta.table.filter  <- betas[(rownames(betas) %in% names(selected.cpgs)),]
```

Analysis

Settings

```
perplexity <- 30
iterations <- 10000
theta <- 0
dimension <- 2
```

Run tSNE

```
betas.sd <- beta.table.filter
betas.sd.centr <- t(scale(t(betas.sd),scale=FALSE))
sv <- svds(betas.sd.centr,k=10)
scores <- t(betas.sd.centr) %*% sv$u
set.seed(01122016)
res <- Rtsne(scores,
             dims=dimension,
             pca=FALSE,
             max_iter=iterations,
             theta=theta,
             perplexity=perplexity)
```

Plot tSNE

```
#Prepare data frame for plotting
dataToPlot <- data.frame(X=res$Y[,1], Y=res$Y[,2])
dataToPlot$samples <- colnames(beta.table.filter)
dataToPlot$group <- mapvalues(dataToPlot$samples,
                              from=annotations$txt_idat,
                              to=annotations$group)
dataToPlot$ID <- mapvalues(dataToPlot$samples,
                           from=annotations$txt_idat,
                           to=annotations$ID)
dataToPlot$group.color <- ''
colors.vec <- refgroup_color

act.plot <- ggplot(dataToPlot, aes(X,Y)) +
  geom_point(aes(color=factor(group),
                 text=paste(paste("ID ", ID),
                            paste("sentrix: ", samples),
                            paste("Group: ", group),sep="<br>")),
```

```
              size=4,
              alpha=0.8) +
  labs(colour="Annotation") +
  coord_equal() +
  xlim(min(dataToPlot$X)-5,
       max(dataToPlot$X)+5)+
  ylim(min(dataToPlot$Y)-5,
       max(dataToPlot$Y)+5) +
  theme_Publication()

ggsave("tsne_plot.pdf",act.plot,width=30, height=30)
```

DMR analysis

DMR analysis

The analysis for differential methylated regions was done with the following packages and settings.

The used R version was: R version 4.0.3 (2020-10-10) – "Bunny-Wunnies Freak Out"

During the analysis the following packages are used:

```
library(openxlsx)
library(parallel)
library(amap)
library(ggplot2)
library(gtable)
library(gridExtra)
library(RColorBrewer)
library(plyr)
library(Rtsne)
library(RSpectra)
library(plotly)
library(dplyr)
library(purrr)
library(siggenes)
library(ggrepel)
library(IlluminaHumanMethylationEPICanno.ilm10b2.hg19)
data("IlluminaHumanMethylation450kanno.ilmn12.hg19")
data("Other")
data("Locations")
data("Islands.UCSC")
```

Preprocessing

Loading the methylation data

```
beta_values.GPL13534 <- read.table('GPL13534_matrix_processed.txt', sep='\t', header=TRUE)
beta_values.GPL13534 <- beta_values.GPL13534[,!grepl('Detection',
                                                      colnames(beta_values.GPL13534))]

beta_values.GPL21145 <- read.table('GPL21145_matrix_processed.txt',sep='\t', header=TRUE)
beta_values.GPL21145 <- beta_values.GPL21145[,!grepl('Detection',
                                                      colnames(beta_values.GPL21145))]

betas.tmp <- cbind(beta_values.GPL13534, beta_values.GPL21145[,-1])

betas <- data.matrix(betas.tmp[,-1])
rownames(betas) <- betas.tmp$ID_REF
colnames(betas) <- gsub('X','', colnames(betas))
```

Loading the annotation data

```
annotation_data <- read.table('annotation.txt', sep='\t', header=TRUE)

groups <- levels(as.factor(annotation_data$group))
colors <- unlist(lapply(groups,
                        function(group) {
                          annotation_data$group_color[annotation_data$group == group][1]
                        }))

annotations <- data.frame(
  txt_idat=annotation_data$idat,
  ID=annotation_data$sample,
  group=annotation_data$group_color
)
```

DMR analysis

Analysis CIC vs EFT CIC

```
makeDMRoutput(allbeta, annotations, path.out="EFT_CIC", control = "EFT_CIC")
```

Analysis CIC vs SBRT CIC

```
makeDMRoutput(allbeta, annotations, path.out="SBRCT_CIC", control = "SBRCT_CIC")
```

DMR analysis helper methods

Helper method for the analysis.

```
makeDMRoutput <- function(allbeta,annotation_data,path.out="./tmp"){

  beta.df <- as.data.frame((allbeta))

  path.out.beta <- paste0(path.out,"/beta/")
  dir.create(path.out.beta, showWarnings = T)

  group.list <- actCases$group
  betaCriteria.table <- apply(allbeta, 1, mad)
  selected.cpgs <- sort(betaCriteria.table, decreasing=TRUE)[1:100000]

  beta.filtered <- allbeta[(rownames(allbeta) %in% names(selected.cpgs)),]

  ########################################################
  ####### Perform analysis for promoter cpgs       #######
  ########################################################

  beta.filteredOnlyPromoter <- beta.filtered[rownames(beta.filtered) %in%
                                             promoterAssosiatedCpgs,]

  promoterAssosiatedCpgs <- cpg.other@rownames[grepl("Promoter",
                                                     cpg.other@listData[[12]]) &
                                               !grepl("Cell", cpg.other@listData[[12]])]
```

```r
M.filteredOnlyPromoter <- log2(beta.filteredOnlyPromoter/(1-beta.filteredOnlyPromoter))
control_pheno <- levels(factor(group.list))
dmp <- dmpFinder(dat = as.matrix(M.filteredOnlyPromoter),
                 pheno = group.list,
                 type = "categorical",
                 shrinkVar = T)

#### Check what is used as Control ####
if(is.null(control)){
  volcano_htitle<-paste0(control_pheno[2],"_vs_",control_pheno[1])
}else{
  if(control_pheno[1]==control){
    volcano_htitle<-paste0(control_pheno[2],"_vs_",control_pheno[1])
  }else{
    dmp$intercept<-dmp$intercept*-1
    volcano_htitle<-paste0(control_pheno[1],"_vs_",control_pheno[2])
  }
}

dmp$cpg<-rownames(dmp)

gene.table <- as.data.frame(sort(table(sig.cpgs$gene),decreasing=TRUE))
colnames(gene.table) <- c("gene","count")
gene.table$gene <- as.character(gene.table$gene)
gene.table$countGene <- as.numeric(mapvalues(gene.table$gene,
                                             from=rownames(cpgsPerGene),
                                             to=cpgsPerGene,
                                             warn_missing=FALSE))
gene.table$ratioGene <- gene.table$count/gene.table$countGene
gene.table <- gene.table[order(gene.table$ratioGene,decreasing=TRUE),]

test <- gene.table[gene.table$ratioGene>=.2,]
test <- test[!is.na(test$ratioGene),]

genes <- test$gene
sort(genes)

results <- map(genes, function(gene) {
  act.gene <- cpg.other@rownames[grepl(gene,cpg.other@listData$UCSC_RefGene_Name)]
  print(paste("cpgCount: ",length(act.gene)))
  used.cpgs <- (rownames(allbeta) %in% act.gene) &
    (rownames(allbeta) %in% promoterAssosiatedCpgs)
  print(paste("cpgPromotor: ",sum(used.cpgs)))

  out.name <- paste(path.out.beta,"/",
                    act.name,"_",
                    volcano_htitle,"_",
                    gene,"_(",
                    sum(used.cpgs),
                    ")_promotor_only",
                    sep="")

  if (sum(used.cpgs)<2) {
```

```r
    used.cpgs <- (rownames(allbeta) %in% act.gene)
    out.name <- paste(path.out.beta,"/",
                      act.name,"_",
                      volcano_htitle,"_",
                      gene,"_(",
                      sum(used.cpgs),
                      ")_complete",
                      sep="")
  }

  beta.gene <- as.data.frame(allbeta[used.cpgs,])

  data.test <- reshape2::melt(t(beta.gene))
  colnames(data.test) <- c("txt_idat","cpg","beta")
  data.test$txt_idat <- as.character(data.test$txt_idat)
  data.test$cpg <- as.character(data.test$cpg)
  data.test$group  <- mapvalues(data.test$txt_idat,
                               from=actCases$txt_idat,
                               to=actCases$group)
  if (sum(used.cpgs)>1) {
    byGroup <- split(data.test,data.test$group)
    test.res <-  t.test(byGroup[[1]]$beta,byGroup[[2]]$beta)
    res.gene <- data.frame(id=actCases$ID,
                          group=actCases$group,
                          means=colMeans(beta.gene[,]))
    p.gene <- ggplot(res.gene,
                     aes(x=group, y=means, fill=actCases$group)) +
      geom_boxplot() +
      guides(fill=FALSE)  +
      ggtitle(gene) +
      ylab("Mean methylation (mean beta-values)") +
      xlab("") +
      theme_bw() +
      theme(axis.text.x = element_text(angle = 90, hjust = 1))

    ggsave(paste(out.name,".png",sep=""),p.gene,height=8)

    data.frame(gene=gene,
               cpgs.complete=length(act.gene),
               cpgs.promoter=sum((rownames(allbeta) %in% act.gene) &
                                   (rownames(allbeta) %in% promoterAssosiatedCpgs)),
               mean.group1=test.res$estimate[1],
               mean.group2=test.res$estimate[[2]],
               mean.diff=test.res$estimate[1]-test.res$estimate[2],
               p.value=test.res$p.value,
               CI.lower=test.res$conf.int[1],
               CI.upper=test.res$conf.int[2])
  } else {
    data.frame(gene=gene,
               cpgs.complete=length(act.gene),
               cpgs.promoter= length((rownames(allbeta) %in% act.gene) &
                                       (rownames(allbeta) %in% promoterAssosiatedCpgs)),
               mean.group1=NA,
```

```r
                 mean.group2=NA,
                 mean.diff=NA,
                 p.value=NA,
                 CI.lower=NA,
                 CI.upper=NA)
  }
})
results <- do.call(rbind, results)
write.xlsx(results[order(results$p.value),],
           paste0(path.out,"/",
                  act.name,"_",
                  volcano_htitle,"_onlyPromoter_genes.xlsx"))
write.xlsx(actCases,paste0(path.out,"/",
                           act.name,"_",
                           volcano_htitle,"_samples.xlsx"))
write.xlsx(sig.cpgs,paste0(path.out,"/",
                           act.name,"_",
                           volcano_htitle,"_sig_promoter_cpgs.xlsx"))

### Map genes with CpGs
geneMapping <- data.frame(cpg=cpg.other@rownames,
                          gene=cpg.other@listData$UCSC_RefGene_Name,
                          stringsAsFactors=FALSE)
geneMapping$chrom <- mapvalues(geneMapping$cpg,
                               from=cpg.locations@rownames,
                               to=cpg.locations@listData$chr)
geneMapping$geneShort <- unlist(lapply(geneMapping$gene,
                                       function(x) strsplit(x,";")[[1]][1]))
cpgsPerGene <- table(geneMapping$geneShort)

###Filter sig cpgs
sig.cpgs <- dmp[dmp$qval < 0.05,]
sig.cpgs$cpg <- rownames(sig.cpgs)

geneMapping.filter <- geneMapping[geneMapping$cpg %in% sig.cpgs$cpg,]
sig.cpgs$gene <- mapvalues(sig.cpgs$cpg,
                           from=geneMapping.filter$cpg,
                           to=geneMapping.filter$geneShort)
sig.cpgs$chrom <- mapvalues(sig.cpgs$cpg,
                            from=geneMapping.filter$cpg,
                            to=geneMapping.filter$chrom)
sig.cpgs <- sig.cpgs[!is.na(sig.cpgs$gene),]


############################ Volcano_plot ################
dmp$significant<-dmp$qval<0.05
dmp <- dmp[order(dmp$qval),]
dmp$gene <- mapvalues(dmp$cpg,sig.cpgs$cpg,sig.cpgs$gene,warn_missing = F)
dmp$gene[dmp$cpg==dmp$gene] <- NA

top_up <- dmp[order(dmp$intercept,dmp$qval,decreasing = T),]
top_down <- dmp[order(dmp$intercept,dmp$qval,decreasing = F),]
label_cpgs <- c(top_up$cpg[1:50],top_down$cpg[1:50])
```

```r
dmp$label <- ifelse(dmp$cpg%in%label_cpgs,dmp$gene,NA)

minfi_volcano_plot <- ggplot(data=dmp,
                             aes(x=intercept,
                                 y=-log10(qval),
                                 col=significant,
                                 label=label)) +
  geom_point() +
  scale_color_manual(values = c("TRUE" = "red", "FALSE" = "black")) +
  geom_text_repel() +
  ggtitle(paste0(volcano_htitle,collapse = "_vs_")) +
  theme_minimal()
minfi_volcano_plot

ggsave(paste0(path.out,"/",
              volcano_htitle,"_promoter_minfi_volcano_plot.pdf"),
       minfi_volcano_plot, height=8, width=6)

########################################################
####Perform analysis for all cpgs #####################
########################################################
M.all<-log2(allbeta/(1-allbeta))
dmp <- dmpFinder(M.all, pheno=group.list, type="categorical",shrinkVar = T)
dmp$cpg<-rownames(dmp)

#### test_vs_control ####

volcano_htitle<-paste0(control_pheno[2],"_vs_",control_pheno[1])

write.xlsx(dmp,paste0(path.out,"/",volcano_htitle,"dmp_allCpgs.xlsx"))

geneMapping <- data.frame(cpg=cpg.other@rownames,
                          gene=cpg.other@listData$UCSC_RefGene_Name,
                          stringsAsFactors=FALSE)
geneMapping$chrom <- mapvalues(geneMapping$cpg,
                               from=cpg.locations@rownames,
                               to=cpg.locations@listData$chr)
geneMapping$geneShort <- unlist(lapply(geneMapping$gene,
                                       function(x) strsplit(x,";")[[1]][1]))

cpgsPerGene <- table(geneMapping$geneShort)

sig.cpgs <- dmp[dmp$qval < 0.05,]
sig.cpgs$cpg <- rownames(sig.cpgs)

geneMapping.filter <- geneMapping[geneMapping$cpg %in% sig.cpgs$cpg,]

sig.cpgs$gene <- mapvalues(sig.cpgs$cpg,
                           from=geneMapping.filter$cpg,
                           to=geneMapping.filter$geneShort)
sig.cpgs$chrom <- mapvalues(sig.cpgs$cpg,
                            from=geneMapping.filter$cpg,
                            to=geneMapping.filter$chrom)
```

```r
sig.cpgs <- sig.cpgs[!is.na(sig.cpgs$gene),]

unique.cpgs <- sig.cpgs %>% distinct(gene)

table(sig.cpgs$chrom)
gene.table <- as.data.frame(sort(table(sig.cpgs$gene),decreasing=TRUE))
colnames(gene.table) <- c("gene","count")
gene.table$gene <- as.character(gene.table$gene)
gene.table$countGene <- as.numeric(mapvalues(gene.table$gene,
                                             from=rownames(cpgsPerGene),
                                             to=cpgsPerGene,
                                             warn_missing=FALSE))
gene.table$ratioGene <- gene.table$count/gene.table$countGene
gene.table <- gene.table[order(gene.table$ratioGene,decreasing=TRUE),]
test <- gene.table[gene.table$ratioGene>=.1,]
test <- test[!is.na(test$ratioGene),]
if(nrow(test)==0){
  return(NULL)
}
write.xlsx(test,paste0(path.out,"/",volcano_htitle,"_differentMethylatedGenes.xlsx"))

genes <- test$gene
sort(genes)

results <- map(genes, function(gene) {
  act.gene <- cpg.other@rownames[grepl(gene,cpg.other@listData$UCSC_RefGene_Name)]
  print(paste("cpgCount: ",length(act.gene)))
  used.cpgs <- (rownames(allbeta) %in% act.gene) &
    (rownames(allbeta) %in% promoterAssosiatedCpgs)
  print(paste("cpgPromotor: ",sum(used.cpgs)))

  out.name <- paste(path.out.beta,"/",
                    volcano_htitle,"_",
                    gene,"_(",sum(used.cpgs),")_all_cpgs",
                    sep="")

  if (sum(used.cpgs)<2) {
    used.cpgs <- (rownames(allbeta) %in% act.gene)
    out.name <- paste(path.out.beta,"/",
                      volcano_htitle,"_",
                      gene,"_(",sum(used.cpgs),")_complete",
                      sep="")
  }

  beta.gene <- as.data.frame(allbeta[used.cpgs,])
  write.xlsx(beta.gene, paste(path.out.beta,"/",
                              volcano_htitle,"_","betas_",
                              gene,".xlsx",sep=""),
             rowNames=TRUE,
             colNames=TRUE)
  data.test <- reshape2::melt(t(beta.gene))
  colnames(data.test) <- c("txt_idat","cpg","beta")
  data.test$txt_idat <- as.character(data.test$txt_idat)
```

```
  data.test$cpg <- as.character(data.test$cpg)
  data.test$group  <- mapvalues(data.test$txt_idat,
                              from=actCases$txt_idat,
                              to=actCases$group)
  if (sum(used.cpgs)>1) {
    byGroup <- split(data.test,data.test$group)
    test.res <-  t.test(byGroup[[1]]$beta,byGroup[[2]]$beta)
    res.gene <- data.frame(id=actCases$ID,
                           group=actCases$group,
                           means=colMeans(beta.gene[,]))
    p.gene <- ggplot(res.gene,
                     aes(x=group, y=means, fill=actCases$group)) +
      geom_boxplot() +
      guides(fill=FALSE) +
      ggtitle(gene) +
      ylab("Mean methylation (mean beta-values)") +
      xlab("") +
      theme_bw() +
      theme(axis.text.x = element_text(angle = 90, hjust = 1))

    ggsave(paste(out.name,".png",sep=""),p.gene,height=8)

    data.frame(gene=gene,
               cpgs.complete=length(act.gene),
               cpgs.promoter = sum((rownames(allbeta) %in% act.gene) &
                                   (rownames(allbeta) %in% promoterAssosiatedCpgs)),
               mean.group1 = test.res$estimate[1],
               mean.group2 = test.res$estimate[[2]],
               mean.diff=test.res$estimate[1]-test.res$estimate[2],
               p.value=test.res$p.value,
               CI.lower=test.res$conf.int[1],
               CI.upper=test.res$conf.int[2])
  } else {
    data.frame(gene=gene,
               cpgs.complete=length(act.gene),
               cpgs.promoter= length((rownames(allbeta) %in% act.gene) &
                                     (rownames(allbeta) %in% promoterAssosiatedCpgs)),
               mean.group1=NA,
               mean.group2=NA,
               mean.diff=NA,
               p.value=NA,
               CI.lower=NA,
               CI.upper=NA)
  }
})
results <- do.call(rbind, results)

write.xlsx(results[order(results$p.value),], paste0(path.out,"/allcpgs_genes.xlsx"))
write.xlsx(actCases,paste0(path.out,"/",volcano_htitle,"_samples.xlsx"))
write.xlsx(sig.cpgs,paste0(path.out,"/",volcano_htitle,"_sig_all_cpgs.xlsx"))

############################ Volcano_plot ################
dmp$significant <- dmp$qval<0.05
```

```
  dmp <- dmp[order(dmp$qval),]
  dmp$gene <- mapvalues(dmp$cpg,sig.cpgs$cpg,sig.cpgs$gene,warn_missing = F)
  dmp$gene[dmp$cpg==dmp$gene] <- NA


  top_up <- dmp[order(dmp$intercept,dmp$qval,decreasing = T),]
  top_down <- dmp[order(dmp$intercept,dmp$qval,decreasing = F),]
  label_cpgs <- c(top_up$cpg[1:50],top_down$cpg[1:50])

  dmp$label <- ifelse(dmp$cpg%in%label_cpgs,dmp$gene,NA)

  minfi_volcano_plot <- ggplot(data = dmp,
                               aes(x=intercept,y=-log10(qval),
                                   col=significant,label=label)) +
    geom_point() +
    scale_color_manual(values = c("TRUE" = "red", "FALSE" = "black")) +
    geom_text_repel() +
    ggtitle(paste0(volcano_htitle,collapse = "_vs_")) +
    theme_minimal()
  minfi_volcano_plot
  ggsave(paste0(path.out,"/",
              volcano_htitle,"_allCpG_minfi_volcano_plot.pdf"),
       minfi_volcano_plot, height=8, width=6)
}
```
